# Gastric lipomas: a case series and review of a rare tumor

**DOI:** 10.1259/bjrcr.20180109

**Published:** 2019-01-16

**Authors:** Ian W Sullivan, Partha Hota, Chandra Dass

**Affiliations:** 1 Department of Radiology, Temple University Hospital, Philadelphia, PA, USA; 2 Department of Radiology, Brigham and Women's Hospital, Boston, MA, USA

## Abstract

The purpose of the study was to investigate and review the multimodality imaging findings of gastric lipomas. Seven patients with gastric lipomas identified by CT imaging at a single institution between 2003 and 2017 were retrospectively evaluated. Patient demographics, clinical presentation, non-invasive imaging, endoscopic, and pathological findings were recorded.The most common location for gastric lipoma was the gastric antrum (3/7). The mean lipoma size was 2.7 cm ± 0.8 cm. Six out of seven lipomas demonstrated homogenous fat attenuation with mean Hounsfield units (HU) between −80 and −120. A single lipoma measuring −50 HU demonstrated soft tissue septations. In addition to routine CT and MRI, gastric lipomas were diagnosed on the low-dose CT protocols such as coronary calcium scoring, renal stone, and positron emission tomography-CT (PET-CT). Our CT findings corroborate those reported previously. Soft tissue septations visualized in one lesion likely represented post-biopsy changes, adding this etiology to a differential which previously included only ulceration. Cases characterized by MRI are rare in the literature, and our study provides one such example. To our knowledge this study represents the first documentation of gastric lipomas on PET-CT and other low-dose CT imaging protocols.

## Introduction

Gastric lipomas are rare tumors, comprising of only 1–3% of benign stomach tumors.^1^ Most gastric lipomas are found incidentally; however, larger tumors can be symptomatic.^2^ Imaging findings of gastric lipomas are similar to those of more common extra gastric lipomas: Typically well circumscribed oval lesions with homogenous fat density. Management of symptomatic lipomas is traditionally endoscopic excision in small lesions with larger lesions undergoing surgery, although this is debated.^3^ Herein we present findings in a series of seven patients with gastric lipomas identified incidentally on cross-sectional imaging. Of note, three of these lipomas were visualized using low-dose CT protocols previously undocumented in the literature, highlighting the potential utility of low-dose CT as a means of follow-up imaging of gastric lipomas. We also present a case of gastric lipoma visualized on MRI, adding another example to the few documented in the literature.

## Methods and Materials

This study was approved by the university institutional review board and, given its retrospective nature, the requirement for informed consent was waived.

Seven cases of gastric lipoma diagnosed incidentally on CT from 2003 to 2017 were reviewed by a body trained radiologist on a picture archiving and communication system (PACS; iSite, Phillips). Recorded variables on CT imaging included mean diameter (average of the anteroposterior, transverse, and craniocaudal dimension), location within the stomach (cardia, fundus, body, antrum, pylorus), and mean attenuation in Hounsfield units (HU). Volume was estimated using the standard ellipsoid formula, V = (π/6)(length × width × height). The mean attenuation of lesions was measured by placing the largest possible round region of interest while attempting to avoid the wall. One patient underwent MRI, for which *T*_2_ and opposed phase gradient echo imaging characteristics of the lesion were recorded. One patient underwent fludeoxyglucose- positron emission tomography/CT (PET/CT) imaging. These images were fused and viewed with Aquarius iNtuition (Terarecon Inc.), and a mean standardized uptake value (SUV) was obtained. Endoscopic images for this case were also reviewed with a board certified gastroenterologist. Pathology for this case was reviewed with a board certified pathologist.

## Results

Seven patients were included in our study with imaging obtained between 2003 and 2017 (Table 1). Four were female and three were male. The mean age at the time of initial scan was 64 (range 53–76). The indications for CT were abdominal pain (*n* = 4), abdominal mass (*n* = 1), unrelated pre-operative planning (*n* = 1), and coronary calcium scoring (*n* = 1). In each study there were other findings to explain the patient’s symptoms and the gastric lipoma was considered as an incidental finding. Management in all cases was observation.

**Table 1.** Patient demographics and gastric lipoma characteristics

ID	Age/gender	Location	Mean diameter (cm)	Volume (ml)	Mean attenuation (–HU)	Figure	Modality
1	71/F	Pylorus	0.5	5.5	90	1	CT without contrast; MRI (MRCP protocol)
2	68/F	Antrum	2.5	5.3	50 (CT), 60 (PET/CT)	2	CT with IV and oral contrast; PET-CT (low dose)
3	53/M	Body	1.3	Partially visualized	120	3a	CT coronary calcium scoring protocol (low dose)
4	73/F	Body	1.0	2.9	100	3b	CT renal stone protocol (low dose)
5	76/M	Antrum	2.1	2.8	80		CT without contrast
6	62/M	Antrum	1.8	9.9	90		CT with contrast
7	47/F	Fundus	2.8	2.0	100		CT without contrast

HU, Hounsfield units; MRCP, magnetic resonance cholangio pancreatography; PET, positron emission tomography.

**Figure 1.**
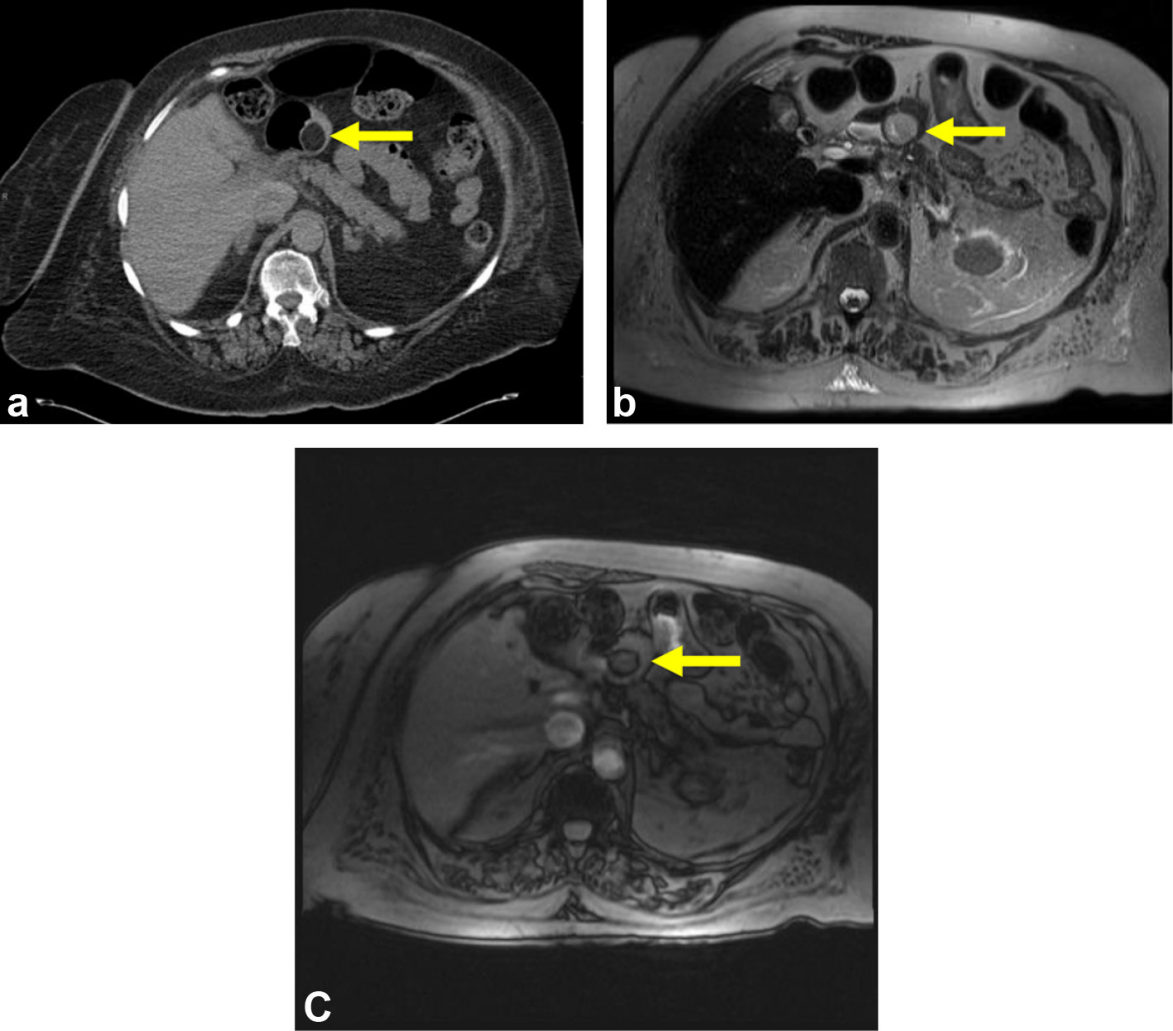
CT and MRI images of a single gastric lipoma. (a) Axial non-contrast CT of the abdomen demonstrates an oval hypoattenuating lesion with HU of −90 within the gastric pylorus, diagnostic for gastric lipoma. (b) Axial MRI T2 W imaging demonstrates signal within the lipoma isointense with the surrounding peritoneal fat. (c) Axial MRI gradient echo out of phase imaging demonstrates chemical shift artifact around the lipoma, diagnostic for its fat content. HU, Hounsfield units; T2 W, *T*_2_ weighted.

**Figure 2.**
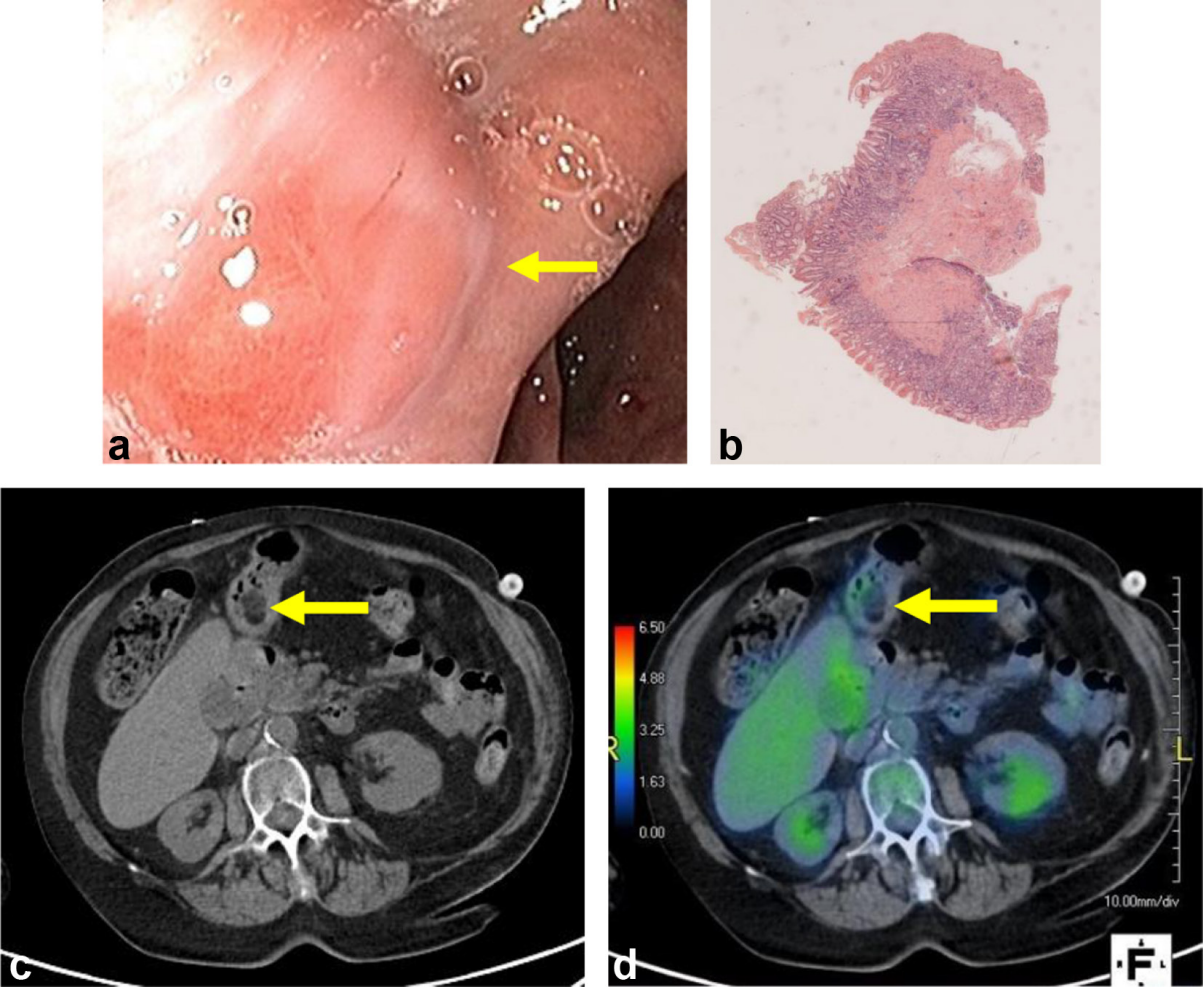
Endoscopic images, histology, CT, and PET-CT fusion images of a single gastric lipoma. (a) Endoscopic image of normal gastric mucosa overlying a convex submucosal mass within the antrum. (b) Mucosal biopsy of the mass demonstrates normal gastric mucosa on histology. No adipose tissue was obtained. (c) 3 years later on axial imaging from a low-dose CT the mass is visualized as a hypoattenuating lesion with HU of −50 consistent with lipoma and thin soft tissue septations, likely representing post-biopsy changes. (d) Fludeoxyglucose-PET fusion with the low-dose CT in Figure 2c demonstrates non-significant uptake of 1.7 SUV within the lipoma. HU, Hounsfield units; PET, positron emission tomography.

The majority of gastric lipomas were found in the antrum (3/7), with the body (2/7) being the next most common location. One lipoma was found in the fundus (1/7) and another was found in the pylorus (1/7). The mean diameter was 2.7 cm (median = 2.4 cm, SD = 0.8 cm) and the mean volume was 4.7 ml (median = 4.1 ml, SD = 2.9 ml). One lesion was incompletely visualized on calcium scoring CT and thus size was not measured. All lesions demonstrated homogenous fat attenuation between −50 and −120 HU (mean = –90, median = –90, SD = 21) (Patient 1, Figure 1a). Soft tissue attenuating septations were demonstrated in a single lipoma measuring −50 HU (Patient 2, Figure 2). Other lipomas did not demonstrate septations. This lipoma was also visualized on PET-CT fusion imaging with low metabolic activity (mean SUV = 1.7). In another patient (Patient 1, Figure 1b,c) the lipoma was also visualized on MRI and demonstrated homogenous isointensity to the adjacent peritoneal fat on *T_2_* weighted (T2 W) Half-Fourier Acquisition Single-shot Turbo spin Echo (HASTE) sequence and bordering Type II chemical shift artifact on opposed-phase gradient echo sequence.

## Discussion

Gastric lipomas are rare with an incidence of 0.029% on autopsy,^4^ and represent 3% of benign gastric masses.^5^ Only 5% of alimentary tract lipomas occur in the stomach, the second rarest location after the esophagus.^5^ The majority of these tumors are confined to the submucosa^2,6^ and are antral in location.^2,6,7^ Although most are solitary, multiple lipomas can occur and 11 cases of diffuse gastric lipomatosis having been described.^4,8^ Due to their rarity there is currently no consensus on sex predilection.

Pathophysiology of gastric lipomas is not entirely understood. Numerous etiologies have been hypothesized, including embryonic misplacement of adipose tissue precursors, chronic irritation, and low-grade infection.^9^ Two cases attributed to the clinical diagnosis of Familial Multiple Lipomatosis have been described, however confirmatory genetic analysis was not performed.^10,11^ Histologically gastric lipomas do not differ from lipomas found in other regions of the body and are composed of mature fat surrounded by a fibrous capsule, characteristics which are reflected on imaging.^6,12^ A differential consideration is well-differentiated gastric liposarcoma. Only 13 cases of gastric liposarcoma have been described and malignant degeneration from gastric lipoma has never been demonstrated.^13,14^ Concomitant adenocarcinoma has been described in four cases.^15–18^ It has been hypothesized that gastric lipomas may predispose the overlying mucosa to repeat erosion and inflammation, factors which are known contributors to gastric cancer risk.^16^ Any departure from classic imaging characteristics in a gastric lipoma may therefore warrant follow-up imaging and/or intervention.

Most gastric lipomas are asymptomatic and are discovered incidentally on autopsy^4,6,12,15^ as Cruveilhier first reported in the mid 19th century.^4,19^ Although, some 50 years earlier, Gourand wrote of a fatty tumor discovered in a patient’s vomitus which he presumed to originate from the stomach.^4,20^ Symptomatic lipomas are often larger than 3 cm and are found in the elderly.^2,6,12^ The most common symptoms are upper abdominal pain and chronic gastrointestinal bleed secondary to ulceration.^2,4^ Less commonly, gastric outlet obstruction may occur and is typically seen with pedunculated lipomas.^21,22^ Ulcerated or intussuscepted lesions may present with acute gastric hemorrhage.^13^

Smaller, asymptomatic gastric lipomas noted incidentally are managed with observation, while larger lipomas which present with clinical symptoms are removed.^3,23,24^ Traditionally this has been accomplished by open or laproscopic partial gastrectomy.^23,25^ While the safety of endoscopic polypectomy for gastrointestinal lipomas with bases measuring less than 2 cm is well established, endoscopic treatment of larger lesions with a broader base is controversial.^26,27^ Simple polypectomy for larger lesions carries a high risk of perforation due to the potential involvement of the underlying muscularis propria.^26^ Endoscopic mucosal resection and endoscopic submucosal dissection are two more novel techniques that have been used to resect large gastric lipomas.^28^ Both involve the injection of a solution of hypertonic saline and epinephrine adjacent to the lesion in order to achieve elevation of the lesion from the muscularis propria. Adequate separation can be verified by endoscopic ultrasound (EUS).^27,29^ Endoscopic mucosal resection is performed with a snare, necessitating the piecemeal excision of larger lesions. Endoscopic submucosal dissection employs an electrocautery knife and allows for *en bloc* dissection, although it requires operator experience and a long procedure time.^28,30,31^ The simplest endoscopic resection technique, unroofing, involves using a snare to resect the pedicle, allowing the remnant of the lipoma to drain passively into the lumen. This technique carries the risk of recurrence,^27,28,30^ although modified methods where subsequent cautery or ligation is performed appear more effective.^27,30^

In 1924, Moore characterized the roentgenological appearance of benign gastric tumors in general as being small and ovoid, with smooth and well-defined contour, projecting into the lumen and often located in the pylorus or body.^32^ Skorneck was the first to pre-operatively diagnose gastric lipoma by fluoroscopy in 1950, citing the tumor's radiolucency.^33^ Fluoroscopy is sensitive for the detection of gastric lipomas, which appear smooth, ovoid, and demonstrate compressibility.^2^ This modality also detects associated ulceration.^2^ The presence of fat radiolucency needed for specific diagnosis is only appreciable in larger lesions, however.^2,6,22^

Meigbow et al^34^ advocated using CT in the diagnosis of gastrointestinal lipoma in 1979 and in 1982 Heiken et al first used this modality to diagnose gastric lipoma.^35^ As on fluoroscopy, the tumors appear smooth and round.^36^ The advantage of CT over fluoroscopy is that the homogenous fat attenuation needed for diagnosis is demonstrable even in smaller lesions, with measured HU of −70 to −120 being diagnostic.^2,6,22^ CT has both superior sensitivity and specificity for diagnosis of gastric lipoma compared to fluoroscopy, endoscopy, and EUS.^7^ Routine endoscopic mucosal biopsy may be insufficient for the diagnosis of gastric lipoma because submucosal tissue is not sampled and therefore false negative biopsy results may be obtained^3,21.^ Many authors caution against the diagnosis of liposarcoma in cases with associated soft tissue density septations or marginal defects identified on CT, as these findings indicate lipoma ulceration.^2,6,13,22^ Gastric liposarcomas are exceedingly rare and based on the few characterized by CT, only well-differentiated tumors demonstrate fatty attenuation, and this is heterogeneous. Higher grade liposarcomas are typically of soft tissue density with cystic areas of necrosis and hemorrhage.^14,37^ Given their characteristic findings and the rarity of potential diagnostic pitfalls, CT is the first diagnostic step in cases of suspected gastric lipoma.^6^

An exception to this rule is in the pediatric population, where radiation exposure is to be minimized, MRI is a viable alternative.^3,13,36,38^ On MRI gastric lipomas demonstrate isointensity to fat on T1 and T2 W imaging with decreased signal on fat suppression.^3,13,36^ On opposed-phase imaging, the fatty mass is also marginated by Type II chemical shift artifact against the water content of the lumen and gastric wall.^36^ T1 fat suppressed sequences are ideal for visualization of involvement of the gastric wall, a useful distinction when deciding between endoscopic *v* *s* surgical resection.^13^ Internal tissue septations appear T1 hypointense and T2 hyperintense when present.^13^ Glucagon may be administered prior to MRI in order to promote gastric distension and reduce peristaltic artifact.^13^

On ultrasound, gastric lipomas appear as homogenously hyperechoic masses marginated by a fibrous hypoechoic capsule.^13,21,29^ Diagnosis may be complicated by copious submucosal fat, the hyperechogenicity of which can cause gastric lipomas to appear relatively hypoechoic.^16^ Abdominal ultrasound can be useful in children and patients of smaller body habitus.^3,13^ EUS is recommended for endoscopic resection planning^29^ because it allows for the localization of the lesion within the five gastric wall layers.^3,29^ EUS also allows for biopsy of the submucosal layer where gastric lipomas typically reside, inaccessible to routine mucosal biopsy.^3^

Many of our findings in our series were consistent with those in the literature. All gastric lipomas were found on imaging incidentally, with lack of symptoms likely due to their relatively small size, as previous studies demonstrated that symptomatic lipomas were often larger than 3 cm^2^. The antrum was the most common location in our case series, which was also consistent with the literature.^2,6,7^ Previous studies had shown attenuations of −70 to −120 HU to be diagnostic for gastric lipoma^2,6,22^ with six out of seven lipomas in our series within this range. A single lipoma (Patient 2, Figure 2) demonstrated an attenuation of −50 HU, likely due to fine internal soft tissue septations.

**Figure 3.**
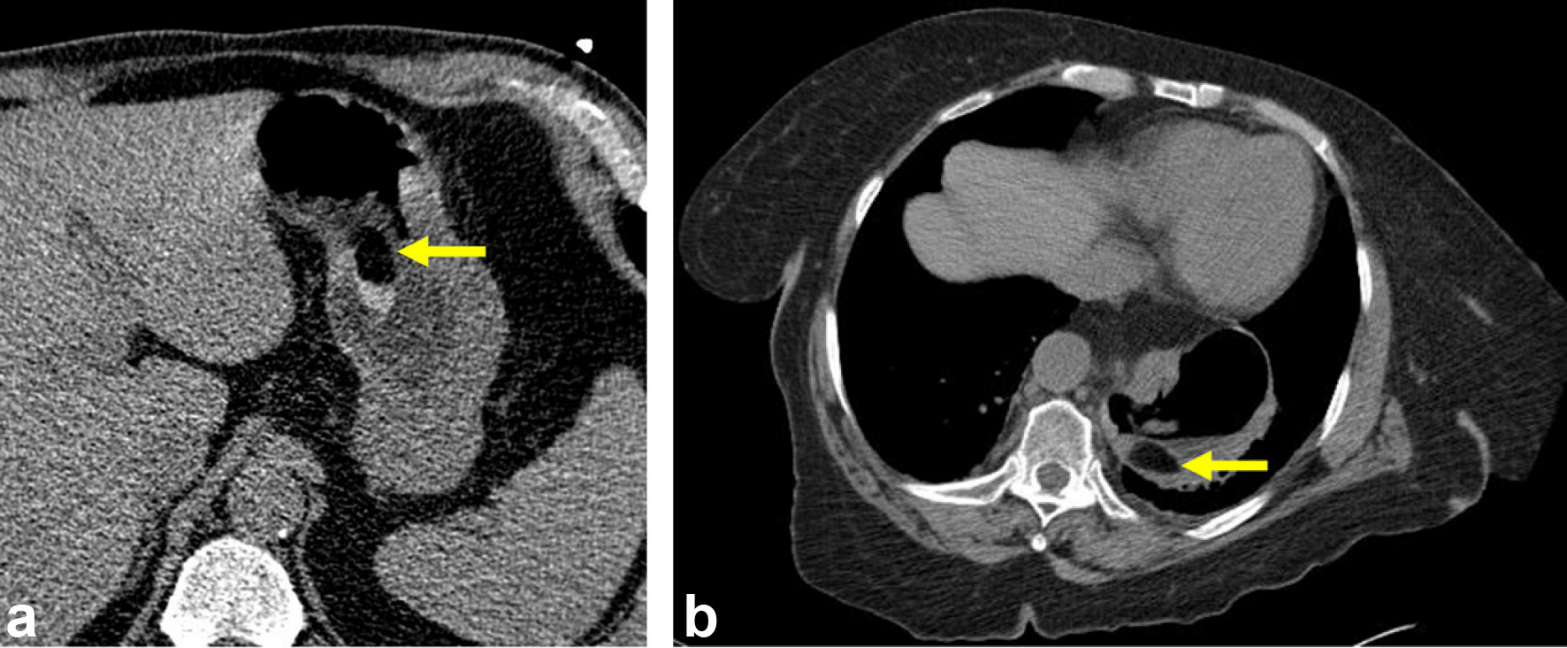
Diagnosis of gastric lipoma by low-dose protocol CT (Figure 2 for low-dose imaging from a PET-CT) (a) Axial imaging from a coronary calcium scoring protocoled CT partially visualizes an oval lesion within the gastric body with HU of −120, diagnostic of gastric lipoma. (b) Axial imaging from a renal stone protocoled CT demonstrates an oval lesion within the gastric body with HU of −100, diagnostic of gastric lipoma. HU, Hounsfield units; PET, positron emission tomography.

To the best of our knowledge, our study presents the first documented case of gastric lipoma visualized on PET-CT (Patient 2, Figure 2). As with the PET appearance of extra gastric lipomas,^39^ the lipoma did not demonstrate any hypermetabolic activity. In addition, the low-dose CT appearance of this lesion was distinctive in that its attenuation was −50 HU and it showed multiple fine septations. 3 years prior the patient had undergone endoscopy for dyspepsia, during which the lesion was biopsied. While the finding of fine soft tissue attenuating septations on imaging has previously by other authors been attributed to ulceration,^2,6,13,22^ no ulcers were found on endoscopy in our patient. We therefore propose that this finding may reflect post biopsy changes. The lipoma was not visualized on imaging prior to biopsy to confirm this hypothesis, however. Endoscopic mucosal biopsy of this lesion was unable to obtain the submucosal lipomatous tissue necessary for diagnosis. In another case the lipoma was visualized on both CT and MRI (Patient 1, Figure 1). The lipoma demonstrated isointensity to that of adjacent peritoneal fat on the T2 W (HASTE) sequence as well as Type II chemical shift artifact on opposed phase gradient echo imaging. This study was protocoled as an MRCP, and thus a fat suppression sequence was not performed. We also present the first documented cases of gastric lipomas diagnosed on low-dose protocoled CTs (Patients 2–4, Figures 2C and 3).

Gastric lipomas are rare tumors for which CT is the diagnostic gold standard, surpassing other imaging studies, endoscopy, and even routine endoscopic biopsy in both sensitivity and specificity. Herein we present a series of cases of gastric lipoma visualized on diagnostic CT, PET-CT, and MRI. In three of our cases gastric lipoma was identified on CT using low-dose protocols, demonstrating the utility of low-dose CT for gastric lipoma follow-up and in patients who cannot tolerate MRI or MRI is contraindicated.

## Learning points

- Gastric lipomas are best diagnosed by CT
- Attenuation from −70 to −120 HU is diagnostic
- Fine soft tissue septations likely indicate ulceration or post-biposy changes; gastric liposarcoma is exceedingly rare
- MRI can be used in children or radiosensitive patients; low-dose CT techniques are also sufficient for diagnosis
- Lipomas smaller than 3 cm are often asymptomatic
- Larger symptomatic lipomas can be resected endoscopically if their base is smaller than 2 cm; resection of lesions with broader bases has traditionally been relegated to surgical resection, although more novel endoscoopic techniques have proven effective
